# Electrotonic transmission in the retinal vasculature: inhibitory role of the diabetes/VEGF/aPKC pathway

**DOI:** 10.14814/phy2.14095

**Published:** 2019-05-13

**Authors:** Maho Shibata, Atsuko Nakaizumi, Donald G. Puro

**Affiliations:** ^1^ Department of Ophthalmology and Visual Sciences University of Michigan Ann Arbor Michigan; ^2^ Department of Molecular and Integrative Physiology University of Michigan Ann Arbor Michigan

**Keywords:** Atypical PKC, diabetic retinopathy, retina, vascular endothelial growth factor

## Abstract

The deleterious impact of diabetes on the retina is a leading cause of vision loss. Ultimately, the hypoxic retinopathy caused by diabetes results in irreversible damage to vascular, neuronal, and glial cells. Less understood is how retinal physiology is altered early in the course of diabetes. We recently found that the electrotonic architecture of the retinovasculature becomes fundamentally altered soon after the onset of this disorder. Namely, the spread of voltage through the vascular endothelium is markedly inhibited. The goal of this study was to elucidate how diabetes inhibits electrotonic transmission. We hypothesized that vascular endothelial growth factor (VEGF) may play a role since its upregulation in hypoxic retinopathy is associated with sight‐impairing complications. In this study, we quantified voltage transmission between pairs of perforated‐patch pipettes sealed onto abluminal cells located on retinal microvascular complexes freshly isolated from diabetic and nondiabetic rats. We report that exposure of diabetic retinal microvessels to an anti‐VEGF antibody or to a small‐molecule inhibitor of atypical PKCs (aPKC) near‐fully restored the efficacy of electrotonic transmission. Furthermore, exposure of nondiabetic microvessels to VEGF mimicked, via a mechanism sensitive to the aPKC inhibitor, the diabetes‐induced inhibition of transmission. Thus, activation of the diabetes/VEGF/aPKC pathway switches the retinovasculature from a highly interactive operational unit to a functionally balkanized complex. By delimiting the dissemination of voltage‐changing vasomotor inputs, this organizational fragmentation is likely to compromise effective regulation of retinal perfusion. Future pharmacological targeting of the diabetes/VEGF/aPKC pathway may serve to impede progression of vascular dysfunction to irreversible diabetic retinopathy.

## Introduction

The constellation of injurious effects of diabetes on the vascular, glial, and neuronal components of the retina ranks as a leading cause of visual impairment. One of the early targets of diabetes‐induced dysfunction is the retina's circulatory system whose ability to adjust blood flow to meet metabolic demand rapidly becomes impaired (Kohner et al. 1995; Ciulla et al. 2002; Clermont and Bursell 2007). In turn, vascular dysregulation impearls the functioning of retinal neurons and glia.

It appears likely that the particular vulnerability of retinal vasculature to diabetes is a consequence of the highly specialized adaptations required to meet the unique challenge of distributing nutrients and oxygen within a tissue whose transparency is essential for light perception (Puro 2012). A distinctive adaptation is the low density of microvessels within the retina (Funk 1997). Yet, even though this sparseness minimizes vascular interference with photons passing to the photoreceptors, the paucity of vessels leaves little functional reserve for maintaining adequate perfusion to meet the stringent metabolic requirements of the retinal neurons. On the other hand, a reserve capacity to adjust blood flow in response to systemic conditions is not needed because of the unique independence of the retinal vasculature from extrinsic control. Namely, its lack of autonomic innervation (Ye et al. 1990) and the tight blood‐retina barrier preclude CNS oversight and exposure of contractile abluminal cells to circulating vasoactive molecules. With the absence of extrinsic input, the retina autonomously regulates its blood flow. Furthermore, effective and efficient autoregulation is facilitated by the retinovasculature's highly interactive electrotonic architecture (Wu et al. 2006; Zhang et al. 2011), which provides the functional infrastructure needed to spatially integrate voltage‐changing vasomotor signals generated at sites throughout the vascular network (Wu et al. 2001; Yamanishi et al. 2006; Puro 2007). However, early in the course of diabetes, electrotonic transmission within retinal microvessels becomes markedly impaired (Nakaizumi et al. 2012), and the ability of the retina to autoregulate blood flow is lost (Kohner et al. 1995; Ciulla et al. 2002; Clermont and Bursell 2007).

The present study builds upon results of earlier electrophysiological analyses demonstrating that diabetes results in the inhibition of gap junction‐dependent communication in the retinal microvasculature (Oku et al. 2001; Nakaizumi et al. 2012; Tien et al. 2016; Roy et al. 2017). More specifically, dual perforated‐patch recordings from microvascular complexes freshly isolated from the retinas of rats with streptozotocin‐induced diabetes revealed a marked attenuation in the efficacy at which voltage spreads axially through microvessels (Nakaizumi et al. 2012). This inhibition of voltage transmission was detected ~5 weeks after the onset of hyperglycemia, which is well before widespread cell death detected in the diabetic rat retina (Mizutani et al. 1996; Gardiner et al. 2007; Barber et al. 2011). The aim of the current study was to elucidate the mechanism by which diabetes inhibits axial transmission in the retinovasculature.

Here, we report that endogenous vascular endothelial growth factor (VEGF) near‐totally accounts for the inhibition of axial transmission observed in diabetic retinal microvessels. In addition, indicative of a key role for atypical PKC isoforms (aPKC), we found that the inhibitory effect of diabetes and VEGF on axial transmission is highly sensitive to a specific, noncompetitive, small‐molecule inhibitor of aPKC. Taken together, our new observations support for a working model in which activation of the diabetes/VEGF/aPKC pathway markedly delimits the spatial dissemination of voltage‐changing inputs within the retinovascular network.

## Materials and Methods

Animal use conformed to the American Physiological Society's Guiding Principles in the Care and Use of Vertebrate Animals in Research and also received approval from the Institutional Animal Care and Use Committee of the University of Michigan. Male Long‐Evans rats were obtained from Charles River (Cambridge, MA, USA). At all times, animals were kept on a 12‐h alternating light/dark cycle and received food and water ad libitum.

### Model of diabetes

Diabetes was induced by one or two intraperitoneal injections of streptozotocin (150 mg kg^−1^ diluted in 0.8 mL citrate buffer) into 5‐week‐old rats that had fasted for 5 h. The diabetic rats used in this study were hyperglycemic for 10.3 ± 1.0 weeks (*n* = 13) and had a blood glucose level of 420 ± 39 mg dL^−1^ immediately retinovessel isolation. Of note, 10 weeks of hyperglycemia are well before vascular cell death is detected in the diabetic rat retina (Mizutani et al. 1996). At the time of sacrifice, the ages of diabetic and nondiabetic rats were not significantly different.

### Microvessel isolation

**Figure 1:**
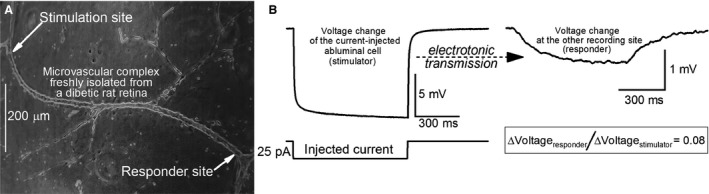
Dual perforated‐patch recordings from a retinal microvascular complex freshly isolated from a diabetic rat. (A) photomicrograph showing a pair of recording pipettes (arrows) at an interpipette distance of 973 *μ*m. (B) left panel, voltage trace during administration via one of the pipettes (stimulator) of a 750‐msec duration 25‐pA hyperpolarizing current. Right panel, voltage trace recorded by the other pipette (responder). To aid visualization, the voltage change at the responding site is displayed at a 5‐fold larger gain.

Using a previously described tissue print procedure (Puro 2012; Puro et al. 2016), we isolated large complexes of microvessels from the retinas of adult rats. In brief, immediately after a rising concentration of carbon dioxide‐induced death, the retinas were removed and placed in solution A, which consisted of 140 mmol/L NaCl, 3 mmol/L KCl, 1.8 mmol/L CaCl_2_, 0.8 mmol/L MgCl_2_, 10 mmol/L Na‐Hepes, 15 mmol/L mannitol, and 5 mmol/L glucose at pH 7.4 with osmolarity adjusted to 310 mosmol L^−1^, as measured by a vapor pressure osmometer (Wescor, Inc., Logan, UT, USA). After removal of adherent vitreous, each retina was cut into quadrants and incubated for 22–26 min at 30°C in 2.5 mL Earle's balanced salt solution supplemented with 0.5 mmol/L EDTA, 6 U papain (Worthington Biochemicals, Freehold, NJ, USA) and 2 mmol/L cysteine; the pH was adjusted to approximately 7.4 by bubbling 5% carbon dioxide. After this incubation, the retinal pieces were transferred to a 60 mm Petri dish containing 5 mL of solution A, and one by one, each retinal quadrant was positioned with its vitreal surface up in a glass‐bottomed chamber containing 1 mL of solution A. Subsequently, each retinal quadrant was gently sandwiched between the bottom of the chamber and a 15 mm diameter glass coverslip (Warner Instrument Corp., Hamden, CT, USA). After ~30 sec, the coverslip was carefully removed; it contained adherent complexes of retinal microvessels, as shown in photomicrographs in Figure 1A and previous publications (Matsushita et al. 2010; Puro 2012). Of note, the correlative anatomical organization of the rat retinal vasculature in vivo and of the retinal microvascular complexes freshly isolated from the rat is available (Puro 2012). A vessel‐containing coverslip was positioned in a recording chamber (volume = 0.5 mL) that could be perfused at 2 mL/min with solutions from a gravity‐fed system using multiple reservoirs.

### Electrophysiology

Experiments were conducted at room temperature within 5 h after isolation of a microvascular complex. Similar to previous studies (Wu et al. 2006; Zhang et al. 2011; Nakaizumi et al. 2012), dual perforated‐patch recordings were used to quantify the efficacy of electrotonic transmission. With earlier analysis demonstrating that diabetes similarly affects electrotonic transmission at all levels of the retinal vascular network (Nakaizumi et al. 2012), this study focused on the secondary retinal arterioles. Of practical importance, the plump “doughnut‐shaped” myocytes encircling the secondary arterioles (Puro 2012) provide relatively easy targets for obtaining stable patch pipette/abluminal seals. Thus, by focusing on these microvessels, it was feasible to obtain sufficient numbers of high‐quality electrophysiological measurements to yield robust, reliable data. Hence, both pairs of recording pipettes were sealed onto a secondary arteriole. Furthermore, the outer diameter of the sampled secondary arteriole was similar at both recording sites. Although some sampled secondary arterioles possessed branching vessels (e.g., Fig. 1A), both recording pipettes were on the targeted secondary arteriole; hence, secondary‐to‐tertiary arteriolar transmission was not assessed. Notable is that branch points within secondary retinal arterioles do not significantly alter the efficacy of electrotonic transmission (Zhang et al. 2011).

Each recording pipette, which had a resistance of 5–10 MΩ and contained a solution consisting of 50 mmol/L KCl, 65 mmol/L K_2_SO_4_, 6 mmol/L MgCl_2_, 10 mmol/L K‐Hepes, 60 *μ*g·mL^−1^ amphotericin B and 60 *μ*g·mL^−1^ nystatin at pH 7.35 and osmolarity at 280 mosmol·L^−1^, was mounted in the holder of a patch‐clamp amplifier (Axopatch 200B, MDS Analytical Technologies, Union City, CA). A piezoelectric‐based micromanipulator (Exfo, Thorlabs, Newton, NJ) facilitated the positioning of the tip of a recording pipette onto to an abluminal cell while the microvasculature complex was viewed at ×400 via an inverted microscope equipped with phase‐contrast optics. As suction was applied to the back end of the pipette, a ≥10 GΩ seal formed. Subsequently, a second recording pipette was sealed onto the same microvessel (Fig. 1A). Interpipette distance was measured from a photomicrograph of the sampled microvascular complex containing the two recording pipettes (Fig. 1A). Data are from recordings in which the access resistances were ≤35 MΩ. Voltages were filtered with a four‐pole Bessel filter, sampled digitally using a DigiData 1440A acquisition system (MDS Analytical Technologies) and stored by a computer equipped with pClamp (version 10, MDS Analytical Technologies), which along with other software (Origin 2017, OriginLab, Northampton, MA, USA), aided with data analysis and graphics display.

For each pair of dual recordings, a 750‐msec hyperpolarizing current step was injected at 3‐sec intervals via one of the two recording pipettes as the membrane potentials of the current‐injected (stimulator) abluminal cell and the other monitored mural cell (responder) were recorded (Fig. 1B). The averaging of voltage traces obtained during 10–30 cycles of current injection facilitated quantification of the voltage changes at the stimulated (Δ*V*_stimulator_) and the nonstimulated (Δ*V*_responder_) sites. Of note, in the retinovasculature there is no significant difference in Δ*V*_responder_/Δ*V*_stimulator_ ratios generated by depolarization or hyperpolarization (Wu et al. 2006; Zhang et al. 2011). Conduction through the bathing solution, rather than through the microvessel, did not contribute to the observed Δ*V*_responder_ since pipette‐to‐pipette transmission was not detected after one of the two recording seals was spontaneously lost or when one of the pipettes was positioned close to, but not sealed onto the microvessel.

### Analysis of Δ*V*_responder_/Δ*V*_stimulator_ measurements

Calculations of the efficacies of axial and radial transmission were based on our previous analysis (Zhang et al. 2011) showing that a voltage generated by injecting current into an abluminal cell is initially transmitted radially to the underlying endothelium, then is transmitted axially through endothelium and finally is transmitted radially to the distantly monitored abluminal cell (responder). Notable is that direct electrotonic communication between adjacent abluminal cells is minimal in retinal microvessels (Zhang et al. 2011). Our previous analysis also showed that voltage decay during axial transmission is described by a first‐order exponential process dependent upon interpipette distance (Zhang et al. 2011). In the present study, we measured Δ*V*_responder_/Δ*V*_stimulator_ ratios at interpipette distances of ≤250 *μ*m (“short” group) and 650–1150 *μ*m (“long” group). By focusing recording efforts on these two groups, it was feasible to measure a sufficient number of Δ*V*_responder_/Δ*V*_stimulator_ ratios to establish reliable values for the mean Δ*V*_responder_/Δ*V*_stimulator_ ratios used in the formula: A^(b−c)/100 *μ*m^ = *d*/*e* where “A” is the efficacy per 100 *μ*m, “b” is the mean interpipette distance for the “long” interpipette distance group, “c” is the mean interpipette distance for the short distance group, “*d*” is the mean Δ*V*_responder_/Δ*V*_stimulator_ ratio for the short interpipette distance group, and “*e*” is the mean Δ*V*_responder_/Δ*V*_stimulator_ ratio for the long distance group. In turn, the percent voltage loss per 100 *μ*m of axial transmission was [(1 − *A*)·100]. As previously detailed (Zhang et al. 2011; Nakaizumi et al. 2012), Δ*V*_responder_/Δ*V*_stimulator_ ratios were also used to calculate the efficacy of radial transmission. In brief, with the aid of commercially available software (OriginLab), the extrapolated Δ*V*_responder_/Δ*V*_stimulator_ ratio at the y‐intercept was computed. With the hypothetical interpipette distance being 0 *μ*m at the y‐intercept, the extrapolated Δ*V*_responder_/Δ*V*_stimulator_ ratio is not affected by axial transmission, but is determined by radial transmissions from stimulated abluminal cell to endothelium and from endothelium to the responder. Hence, the square root of the extrapolated Δ*V*_responder_/Δ*V*_stimulator_ ratio at 0 *μ*m is the efficacy of a radial transmission. From this efficacy, it is straightforward to calculate the percent of voltage lost during a radial transmission.

### Chemicals

The specific inhibitor of atypical PKC, propan‐2‐yl 2‐amino‐4‐(3,4‐dimethoxyphenyl)thiophene‐3‐carboxylate (Titchenell et al. 2013), was a gift from David Antonetti. Other chemicals were from MilliporeSigma (St. Louis, MO) including recombinant rat vascular endothelial growth factor 164 (MilliporeSigma catalog number V3638) and an anti‐VEGF antibody developed in goat using a purified 164 amino acid residue variant of recombinant mouse VEGF (MilliporeSigma V1253; RRID: AB_261846).

### Statistics

Data are given as mean ± SE. Probability was evaluated by Student's two‐tailed *t*‐test, with equal or unequal variance, as appropriate. For comparison of two groups, *P* > 0.05 indicated failure to detect a significant difference. The Bonferroni correction was used to adjust the *P*‐value for significance when >2 groups were compared (Figs. 4 and 6).

## Results

**Figure 2:**
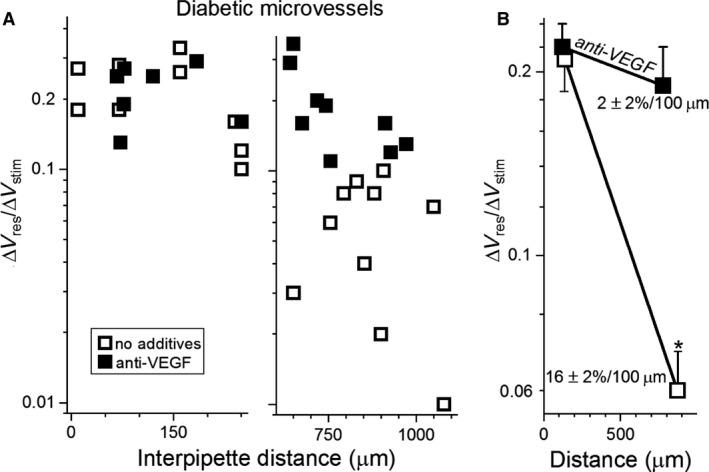
Effect of an anti‐VEGF antibody on electrotonic transmission in diabetic retinal microvessels. (A) Δ*V*_responder_/Δ*V*_stimulator_ ratios obtained at relatively short (left panel) and long (right panel) interpipette distances in the absence or presence for >2.5 h of 3 *μ*g/mL anti‐VEGF. Data points in the absence of the antibody include four in the short interpipette distance group and three in the long group from Nakaizumi et al. (2012). (B) mean Δ*V*_responder_/Δ*V*_stimulator_ ratios plotted at the mean interpipette distances. Voltage decay rates calculated as described in the Materials and Methods are shown. *P = 0.0002.

The aim of this study was to elucidate how diabetes alters the electrotonic architecture of the retinal microvasculature. Previously, simultaneous dual perforated‐patch recordings revealed that the axial spread of voltage through the endothelium is markedly inhibited in diabetic retinal microvessels (Nakaizumi et al. 2012). As a framework for the present study, we hypothesized that vascular endothelial growth factor (VEGF) may play a key role in mediating this diabetes‐induced inhibition of axial transmission. VEGF was of interest since its upregulation is known to play a role in diabetic retinopathy (Antonetti et al. 2012; Jiang et al. 2015; Kida et al. 2017) and gap junction‐dependent intercellular communication in various nonretinal vascular cells can be inhibited by VEGF (Suarez and Ballmer‐Hofer 2001; Thuringer 2004; Nimlamool et al. 2015). To assess the putative role of VEGF, microvessels freshly isolated from diabetic retinas were preexposed for >1 h to an anti‐VEGF antibody (3 *μ*g/mL). Subsequently, Δ*V*_responder_/Δ*V*_stimulator_ ratios were measured via dual recording pipettes (Fig. 2A). In other experiments, dual recordings were also obtained from diabetic microvessels in the absence of the antibody (Fig. 2A). Analysis of the Δ*V*_responder_/Δ*V*_stimulator_ ratios revealed that anti‐VEGF treatment attenuated by 8‐fold (*P* = 0.0002) the rate of voltage decay during axial transmission (Fig. 2B). This robust effect indicates that endogenous VEGF plays a key role in mediating the diabetes‐induced inhibition of axial transmission.

**Figure 3:**
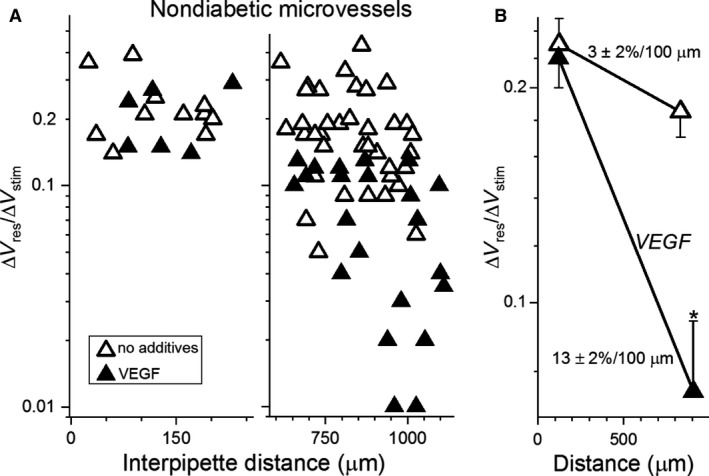
Effect of VEGF on electrotonic transmission in nondiabetic retinal microvessels. (A) Δ*V*_responder_/Δ*V*_stimulator_ ratios at short (left panel) and long (right panel) interpipette distances in the absence or presence for > 1 h of 100 ng/mL VEGF. Data points in the absence of VEGF include five in the short group and two in the long group from Nakaizumi et al. (2012). (B) mean Δ*V*_responder_/Δ*V*_stimulator_ ratios plotted at the mean interpipette distances. Voltage decay rates are shown. *P = 0.0029.

In complementary experiments, we tested the effect of exogenous VEGF on axial transmission in microvessels from nondiabetic retinas. Specifically, prior to measuring Δ*V*_responder_/Δ*V*_stimulator_ ratios, nondiabetic retinal microvessels were preincubated for >1 h in the absence or presence of 100 ng/mL VEGF (Fig. 3A), which is the reported concentration at which there is near‐maximal binding to VEGF receptors (Matsumoto and Claesson‐Welsh 2001; Krilleke et al. 2007). Analysis of the measured Δ*V*_responder_/Δ*V*_stimulator_ ratios showed that treatment with VEGF markedly increased (*P* = 0.0029) the rate of voltage decay during axial transmission (Fig. 3B). Of note, this series of experiments performed under normoglycemic conditions shows that the ability of VEGF to increase the voltage decay rate does not require an elevated concentration of glucose. In addition, this effect was not associated with a change in the recorded membrane potential, which was −44 ± 1 mV in the absence (*n* = 28) and presence of VEGF (*n* = 47). Also of note, the voltage decay rates during axial transmission in diabetic microvessels (Fig. 2B) and in VEGF‐treated nondiabetic microvessels (Fig. 3B) were not significantly different. Thus, exposure of nondiabetic retinal microvessels to exogenous VEGF mimicked the diabetes‐induced inhibition of axial transmission.

To further assess the ability of VEGF to mimic the effect of diabetes on the electrotonic architecture, we asked whether VEGF, like diabetes (Matsushita et al. 2010), fails to significantly alter radial transmission. Indicative that this is in fact the case, analysis of Δ*V*_responder_/Δ*V*_stimulator_ ratios measured in nondiabetic microvessels (Fig. 3A) showed that voltage decay during radial transmission was not significantly altered by VEGF. Namely, in nondiabetic microvessels, voltage loss during radial transmission in the absence of VEGF was 50 ± 2% and in the presence of VEGF, 52 ± 2%. Furthermore, anti‐VEGF treatment of diabetic microvessels did not significantly alter voltage decay during radial transmission; decay was 49 ± 2% in the absence of this antibody and 53 ± 6% in its presence. Based on these observations, we concluded that neither diabetes nor VEGF impairs radial transmission in retinal microvessels. Rather, as observed in diabetic retinal vessels, VEGF selectively inhibits axial transmission.

**Figure 4:**
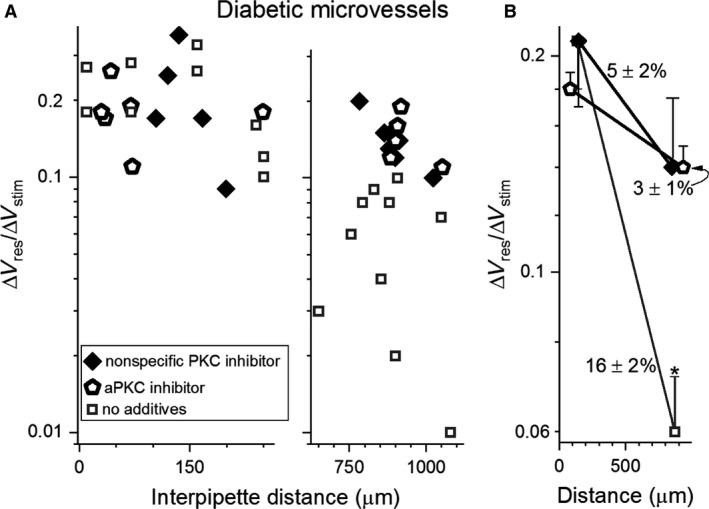
Effect of PKC inhibitors on electrotonic transmission in diabetic retinal microvessels. (A) Δ*V*_responder_/Δ*V*_stimulator_ ratios at short (left panel) and long (right panel) interpipette distances in the absence (data from Fig. 2A) or presence for > 1 h of either the nonspecific PKC inhibitor chelerythrine (1 *μ*mol/L) or 100 nmol/L of a specific aPKC inhibitor. (B) means of the data in A plotted at the mean interpipette distances. Voltage decay rates per 100 *μ*m are show. *P ≤ 0.0022.

To further elucidate the mechanism by which diabetes inhibits axial transmission in retinal microvessels, we postulated that the protein kinase C (PKC) family of enzymes plays a role. PKC was of interest since VEGF can activate PKC (Das Evcimen and King 2007; Titchenell et al. 2012) and in various cell types, PKC activation inhibits gap junction function (Suarez and Ballmer‐Hofer 2001; Thuringer 2004; Nimlamool et al. 2015). To begin to assess the putative role of PKC, we measured Δ*V*_responder_/Δ*V*_stimulator_ ratios in diabetic retinal microvessels exposed for >1 h to the broad‐spectrum PKC inhibitor, chelerythrine (1 *μ*mol/L; Fig. 4A). Analysis of Δ*V*_responder_/Δ*V*_stimulator_ ratios showed that chelerythrine significantly lessened (*P* = 0.0022) the diabetes‐induced inhibition of axial transmission (Fig. 4B).

**Figure 5:**
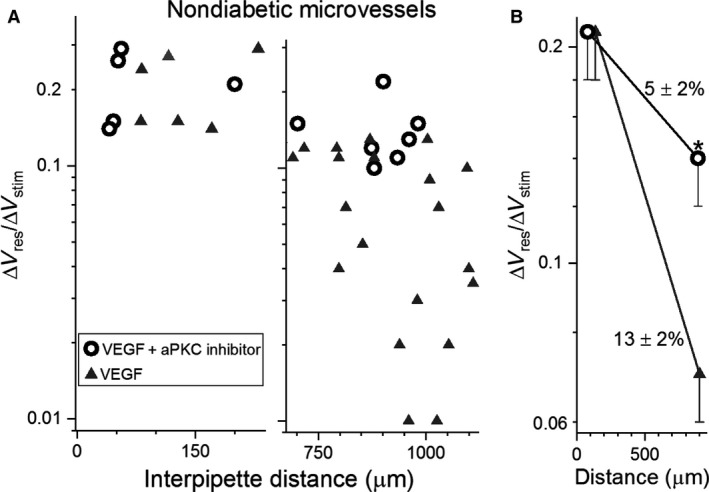
Effect of an inhibitor of aPKC on the VEGF‐induced boost in voltage decay in nondiabetic microvessels. (A) Δ*V*_responder_/Δ*V*_stimulator_ ratios measured at short (left panel) and long (right panel) interpipette distances in nondiabetic microvessels exposed for >2.5 h to 100 ng/mL VEGF in the absence (data from Fig. 3A) or presence of the specific aPKC inhibitor (100 nmol/L) for > 1 h. (B) means of the data in A plotted at the average interpipette distances. Rates of voltage decay per 100 *μ*m are shown. *P = 0.0117.

With emerging evidence that atypical PKC (aPKC) isoforms may be involved in diabetic vascular complications (Wellner et al. 1999; Titchenell et al. 2012; Lin et al. 2018), we also tested the effect of the specific, potent, noncompetitive, small‐molecule inhibitor of aPKC inhibitor, propan‐2‐yl 2‐amino‐4‐(3,4‐dimethoxyphenyl)thiophene‐3‐carboxylate (100 nmol/L). Dual recording experiments showed that exposure of diabetic retinal microvessels to this aPKC inhibitor near‐fully reversed (*P* = 0.0001) the inhibition of axial transmission (Fig. 4). Also indicative of the importance of aPKC, additional recordings demonstrated that exposure of nondiabetic microvessels to the small‐molecule aPKC inhibitor largely prevented the VEGF‐induced inhibition of axial transmission (Fig. 5).

**Figure 6:**
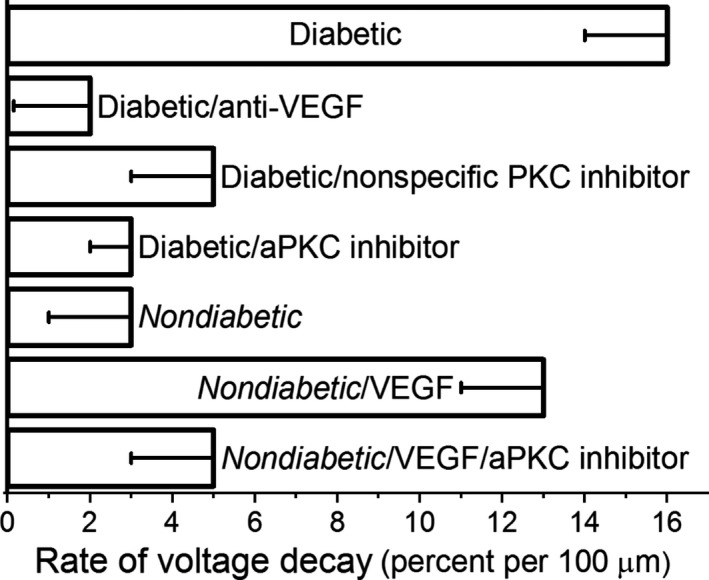
Voltage decay rates during axial transmission in diabetic and nondiabetic retinal microvessels under various experimental conditions. The diabetic group (top row), as well as the nondiabetic/VEGF group (sixth row), had significantly more rapid voltage decay rates than those measured in the other experimental groups.

Taken together, the results of the dual recording experiments summarized in Figure 6 support a pathophysiological scenario in which endogenous VEGF and the activation of aPKC mediate the diabetes‐induced inhibition of axial transmission in the retinovasculature.

## Discussion

This analysis of the electrotonic transmission in retinal microvessels provides evidence that endogenous VEGF, via an aPKC‐dependent mechanism, mediates the diabetes‐induced inhibition of axial transmission. Using dual perforated‐patch recordings from microvessels freshly isolated from the retinas of rats made diabetic by streptozotocin, we demonstrated that exposure to an anti‐VEGF antibody or to a small‐molecule inhibitor of aPKC restored the efficacy of axial transmission to the level observed in nondiabetic retinal microvessels. Conversely, dual recordings also established that by a mechanism sensitive to the aPKC inhibitor, exposure of nondiabetic retinal microvessels to exogenous VEGF mimicked the inhibitory effect of diabetes on axial transmission. This analysis revealed that activation of the diabetes/VEGF/aPKC pathway markedly delimits the spatial dissemination of focally induced voltage changes. For example, calculations using the measured Δ*V*_responder_/Δ*V*_stimulator_ ratios indicate that activation of this pathway boosts voltage loss from 8% to 50% during a 400‐*μ*m axial transmission and from 50% to 99.5% during a 3200‐*μ*m transmission. We posit that this functional fragmentation impairs the ability of the retinovasculature to effectively integrate the many voltage‐changing vasomotor signals (Li and Puro 2001; Wu et al. 2001; Yamanishi et al. 2006; Puro 2007) generated at sites through the vascular network.

The new observation that VEGF alters the electrotonic architecture of the retinal vasculature raises the question as to whether this molecule also inhibits intercellular communication in other vasculatures. Indicative of this, exposure of nonretinal endothelial cells to VEGF can inhibit cell‐to‐cell communication via gap junctions (Suarez and Ballmer‐Hofer 2001; Thuringer 2004; Nimlamool et al. 2015), whose closure is known to modulate angiogenesis (Fang et al. 2011, 2012; Gartner et al. 2012; Okamoto et al. 2014). In addition to altering gap junction function, retinal and nonretinal vasculatures share other responses to VEGF. For example, a common response is increased leakage of serum‐derived angiogenic molecules (Ferrara 1999; Bao et al. 2009; Titchenell et al. 2012). Also, VEGF directly stimulates the growth of neovessels in numerous tissues including the retina (Ferrara 1999; Melincovici et al. 2018). Yet, despite these similarities, VEGF elicits a beneficial adaptive response to hypoxia only in nonretinal tissues (Dor et al. 2002). In the hypoxic retinopathy of diabetes, sickle cell disease or premature birth, endogenous VEGF stimulates the growth of grossly aberrant neovessels that fails to enhance effective retinal perfusion, are prone to vision‐impairing hemorrhages and, by generating tractional forces, can cause blinding retinal detachments. Furthermore, although a VEGF‐induced leakage of angiogenic molecules from nonretinal vessels facilitates a well‐adapted neovascular response (Bao et al. 2009), the VEGF‐triggered breakdown of the blood‐retinal barrier results in edematous swelling that can impair sight by disrupting the retina's highly specialized morphological and functional organization (Antonetti et al. 2012). Additionally, as noted, the diabetes/VEGF/aPKC‐induced restriction in the electrotonic dissemination of voltage‐changing vasomotor inputs is likely to compromise the unique ability of the retina to autoregulate its blood flow. Thus, there is a stark dichotomy in the adaptive effectiveness of VEGF at nonretinal and retinal sites. We postulate that the adaptive role of VEGF in nonretinal tissues has been honed throughout much of mammalian evolution to effectively ameliorate hypoxia. In contrast, with life expectancy having increased only recently for diabetics and others prone to hypoxic retinopathy, there has been insufficient time for evolutionary pressure to tailor the responses of the retina to VEGF so that hypoxia is ameliorated while optimal light detection is maintained.

This analysis of electrotonic transmission in the retinovasculature was based on analysis of freshly isolated microvascular complexes. With this experimental preparation, it is quite feasible to obtain dual perforated‐patch recordings at specific microvascular locations. Furthermore, use of isolated retinal microvessels allowed assessment of the effects of VEGF and inhibitors of PKC in the absence of confounding actions mediated via nonvascular cells. On the other hand, effects on voltage transmission of the numerous vasomotor signals generated within the retina (Puro 2007) were not assessed. Also, because the isolated microvessels were not internally perfused, the effect of intraluminal pressure on electrotonic transmission was not evaluated. Additionally, even though the conduction of electric signals has been intensively studied in nonretinal vasculatures (Figueroa and Duling 2009; Behringer and Segal 2012), the effect of diabetes on voltage transmission in these vascular beds has yet to be explored. Also, future studies of interest include measurement of abluminal VEGF levels on diabetic microvessels, identification of the connexins affected by the diabetes/VEGF/aPKC pathway and determination of this pathway's impact on the autoregulation of retinal blood flow. It is clear that pathophysiological mechanisms characterized in isolated vascular complexes will require in vivo verification, although technical advances are required to allow quantification of electrotonic transmission within the vasculature of the in vivo retina.

The key conclusion of this study is that the mechanism by which diabetes inhibits axial transmission in the retina microvasculature is near‐totally due to the actions of VEGF and aPKC. Further, the results show for the first time that exposure to anti‐VEGF antibodies, which are now commonly used in the treatment of complications of diabetic retinopathy (Apte et al. 2019), increases in the efficacy of axial transmission in diabetic retinovessels. This raises the possibility that this previously unappreciated action of anti‐VEGF may play a role in its therapeutic impact.

Our experiments using nondiabetic microvessels under normoglycemic conditions demonstrate that an elevated concentration of glucose is not required for the VEGF‐induced inhibition of transmission. This finding supports the possibility that the VEGF/aPKC pathway may also impair electrotonic transmission in nondiabetic conditions, such as sickle cell retinopathy, in which VEGF production is induced by retinal hypoxia. On the other hand, in contrast to the pathophysiological importance of VEGF in the adult retina, the minimal amount of this growth factor in the mature retina (Penn et al. 2008; Shimada et al. 2009) suggests a minor physiological role.

In conclusion, results from our dual perforated‐patch recordings indicate that early in the course of diabetes, a mechanism dependent on VEGF and aPKC inhibits axial transmission in retinal microvessels. In this way, the electrotonic architecture of the retinovasculature is switched from a highly interactive global system to a functionally balkanized complex. We proposed that the organizational disruption induced by the diabetes/VEGF/aPKC pathway plays a previously unappreciated role in the blood flow dysregulation that occurs early in the course of diabetes. In the future, pharmacological manipulation of the diabetes/VEGF/aPKC pathway may provide a new strategy for preventing perturbations in retinovascular function from progressing to irreversible sight‐impairing diabetic retinopathy.

## Conflict of Interest

No conflicts of interest, financial or otherwise, are declared by the authors.
